# Exploring quality improvement for diabetes care in First Nations communities in Canada: a multiple case study

**DOI:** 10.1186/s12913-023-09442-3

**Published:** 2023-05-09

**Authors:** Meghan Fournie, Shannon L. Sibbald, Stewart B. Harris

**Affiliations:** 1 Western University, London, ON N6G 1G7 Canada; 2 Faculty of Health Sciences, Western University, London, ON Canada; 3 The Schulich Interfaculty Program in Public Health, Schulich School of Medicine & Dentistry, Western University, London, ON Canada; 4 Department of Family Medicine, Schulich School of Medicine & Dentistry, Western University, London, ON Canada

**Keywords:** Chronic disease, Diabetes, Indigenous, Primary healthcare, Quality improvement, Qualitative, Case Study

## Abstract

**Background:**

Indigenous peoples in Canada experience higher rates of diabetes and worse outcomes than non-Indigenous populations in Canada. Strategies are needed to address underlying health inequities and improve access to quality diabetes care. As part of the national FORGE AHEAD Research Program, this study explores two primary healthcare teams’ quality improvement (QI) process of developing and implementing strategies to improve the quality of diabetes care in First Nations communities in Canada.

**Methods:**

This study utilized a community-based participatory and qualitative case study methodology. Multiple qualitative data sources were analyzed to understand: (1) how knowledge and information was used to inform the teams’ QI process; (2) how the process was influenced by the context of primary care services within communities; and (3) the factors that supported or hindered their QI process.

**Results:**

The findings of this study demonstrate how teams drew upon multiple sources of knowledge and information to inform their QI work, the importance of strengthening relationships and building relationships with the community, the influence of organizational support and capacity, and the key factors that facilitated QI efforts.

**Conclusions:**

This study contributes to the ongoing calls for research in understanding the process and factors affecting the implementation of QI strategies, particularly within Indigenous communities. The knowledge generated may help inform community action and the future development, implementation and scale-up of QI programs in Indigenous communities in Canada and globally.

## Background

The history of colonization, racism, and social exclusion are recognized as key social determinants of health affecting the wellbeing of Indigenous peoples in Canada [1]. Together, these create jurisdictional and geographic barriers to accessing health care services [2], and barriers to culturally safe and appropriate care [3, 4]. The inequities in access to health care and the poorly structured health care services in Indigenous communities have been associated with worse health outcomes, especially for those living with diabetes [5, 6]. Strategies are needed to address underlying health inequities and improve access to high-quality diabetes care services for Indigenous peoples in Canada [7, 8].

Team-based strategies, such as quality improvement collaborative (QIC) programs, have been found to be the most effective at facilitating improvements in chronic disease care within primary care settings [9]. In Canada, research on the effectiveness of such strategies in primary care settings have shown improvements in diabetes care and health outcomes [10–13]. However, multiple systematic reviews on the effectiveness of QIC programs have shown mixed results [14–16]. This difference in results has been attributed to the highly context-dependent nature of quality improvement (QI) efforts [14, 15, 17]. Most studies have focused on evaluating their impact on anticipated clinical outcomes by using controlled or before-after study designs that do not capture the dynamic nature of QI, nor the contexts that lead to success or failure [18, 19]. As a result, there are increasing calls for research on contextual factors that influence QI [20, 21].

Implementation research is one way to address these calls by seeking to understand QI processes and factors affecting implementation [22]. Globally, there has been limited research on the implementation of health services and programs with Indigenous communities [23, 24]. The purpose of this study was to explore primary healthcare teams’ process of developing and implementing QI strategies aimed at improving the quality of diabetes care in First Nations communities in Canada. This research is a sub-study of the TransFORmation of IndiGEnous PrimAry HEAlthcare Delivery (FORGE AHEAD) Research Program and includes secondary analysis of data collected as part of the larger research program.

### The FORGE AHEAD Research Program

The FORGE AHEAD Research Program embedded community-based participatory research principles to partner with 11 First Nations communities across Canada with the goal of improving diabetes care and access for Indigenous communities. The design of the FORGE AHEAD Research Program and how community-based participatory research principles were enacted have been published elsewhere [25]. The program consisted of two 18-month QIC programs based on the Institute for Healthcare Improvement’s Breakthrough Series [26]. A clinical team of primary healthcare professionals in the community participated in the clinical-based program. Simultaneously, a community team consisting of community members and diabetes program staff participated in the community-based program. The study described in this manuscript focuses on the QI process of two clinical teams that participated in the clinical-based QIC program from 2014 to 2016.

Based on the Model for Improvement and Plan-Do-Study-Act (PDSA) methodology, the clinical team engaged in an iterative QI process throughout the program of identifying priority areas affecting the quality of diabetes care and outcomes, developing community-driven QI strategies, and testing and evaluating them in practice [27]. The team participated in a series of three workshops over the course of the 18-month program to learn about evidence-based practices for improving diabetes care and outcomes. During the workshops, team members were trained in the QI methodology and were provided dedicated time during breakout sessions to discuss and plan practice changes. Between the workshops, teams continued to develop and implement QI strategies. A community facilitator led both teams throughout the program and facilitated breakout sessions and team meetings. Research team facilitators helped to moderate team breakout sessions at the workshops and conducted support calls in between workshops to discuss the teams’ progress and help them apply the QI methodology. The program integrated two tools designed to help teams identify priority areas to target for QI and evaluate the success of their QI strategies: a clinical readiness consultation report and a diabetes registry and surveillance system. The clinical readiness consultation report summarized information collected from the team on the existing diabetes care delivery systems and services within the community [25, 28]. The diabetes registry and surveillance system stored a list of adults with diabetes in the community and their clinical information.

## Methods

Utilizing community participatory research principles and case study methodology, this study aimed to answer the following questions:

1. How was knowledge and information used by the clinical teams to inform their QI process for improving diabetes care?
2. How were the clinical teams’ QI processes shaped by the context of primary care services within First Nations communities in Canada?
3. What factors supported or hindered the clinical teams’ diabetes QI process?

The researchers ensured collaboration with the two community partners and discussed the research design and questions with the designated community representatives (healthcare leadership, QI team leads, community members) to ensure the study was relevant and meaningful to the community. Over multiple meetings, the researcher met with the community representatives to discuss their individual case findings to provide insight from those directly involved in the program. Qualitative multiple case study methodology guided data collection and analysis [29]. Case study methodology was ideally positioned to give new insights into the process of implementing changes in practice to improve the quality of care, and greater attention to detail on QI efforts within diverse community contexts [29, 30].

### Case selection

Two clinical teams that participated in the clinical-based QIC program were selected based on diversity in the characteristics of the community’s remoteness level, governance, and geographical location. This case selection was based on factors that affect First Nations communities’ health care services and allowed for examining the complexity of diabetes QI activities occurring across diverse settings [31].

### Data collection and procedures

A secondary analysis of multiple qualitative data sources (Table 1) was conducted to capture a holistic understanding of clinical teams’ diabetes QI process.

**Table 1:** Summary of Data Sources

Data Source	Description	Quantity of Data
Observation Field Notes	Observation of workshop breakout sessions • Workshop 1: 4 Breakout Sessions (approx. 4 h) • Workshop 2: 2 Breakout Sessions (approx. 2 h) • Workshop 3: 2 Breakout Sessions (approx. 2 h)	32 field and reflective notes (16 per team)
Team Member Interviews	End of program interviews (approx. 1 h) with clinical QI team members	7 interview transcripts (4 Team West interviews, 3 Team East interviews)
QI Activity Documentation	Documentation of diabetes QI activities on PDSA cycle templates	25 pages (10 pages from Team West, 15 pages from Team East)
Implementation Support Notes	Documentation of implementation support	44 pages (21 pages from Team West; 23 pages from Team East)

1. **Observation Field Notes**: A member of the research team recorded field notes during breakout sessions at the three workshops. They recorded descriptions of the activities, discussions, individual and group actions, and the roles of program facilitators.
2. **Team Member Interviews**: Team members were purposefully selected for maximum variation based on their professional role and community membership. One-hour interviews were conducted at the end of the program with seven team members from the two communities included in this study. Interviews were open-ended and flexible and included questions on the experience with program activities, facilitators’ role, existing barriers to participation, perceptions of the program’s impact, and their QI strategies. Audio-recordings were transcribed verbatim by an external transcription company and verified by a research team member.
3. **QI Activity Documentation**: The PDSA document that teams used to keep track of the strategies they tested to improve care, including outcomes and challenges, was collected at the end of the program.
4. **Implementation Support Notes**: After support calls with the teams, the research facilitators documented their discussions and personal observations.

### Data analysis

Data sources were analyzed iteratively and triangulated to generate a holistic perspective of the context, activities, and experiences of each team. Each case was analyzed individually and followed by a cross-case analysis of categories and themes. Memo and reflexive notes were taken throughout data analysis. In the first phase, the researcher (MF) immersed themself in the data by reading each data source several times and listening to interview recordings to gain insight into the research environment and context of the interview. Each data source was inductively analyzed and a combination of descriptive, process, and in vivo codes were developed. After initial inductive coding, the researcher returned to the data to examine possible theoretical explanations and create interpretative and theoretical codes. The Promoting Action on Research Implementation in Health Services (PARIHS) framework was used as a conceptual device to help understand the emerging themes and the complexity of implementation [32]. The framework represents *successful implementation* of evidence into practice as a dynamic, complex interaction between three core elements; the nature and type of *evidence*, the *context* in which implementation takes place, and the way in which implementation is *facilitated* [33]. Multiple rounds of reorganizing and re-contextualizing the data were performed by assessing the codes for frequency and analytical strength. Lastly, analysis proceeded to understand the patterns, similarities, and differences across the two teams. Throughout data analysis, the researchers met to discuss initial codes and emerging findings. A summary of initial within-case findings were sent to the community representatives. Later, the researcher met with them by teleconference to discuss the findings. The community representatives provided additional perception into their experiences with and connections between emerging categories and themes. Afterward, the researcher returned to the data and reflected on any new insights into the data that the community representatives provided. In particular, the community representatives gave further insight into the structure of the primary care services and clarity around the QI strategies that they implemented within their community. These insights were reflected in the reported findings. The results of this study were shared back with the communities and the final results were presented at community meetings.

## Findings

A summary description of the teams and communities is provided in Table 2. The names and location of the communities have been de-identified to comply with community research agreements. Over the course of the clinical-based QIC program, Team West consisted of six team members from a variety of different healthcare professional roles within the primary care system. The structure of primary care services and delivery in the community consists four main groups, all residing within a large health centre in the community: (1) a primary care clinic, (2) diabetes and health education programs, (3) homecare, and (4) community and public health programs. The community is located within 100 km of a large urban centre.

**Table 2:** Community and Clinical Team Description

	Community Description			Clinical Team Description		
Team Name	Health Service Funding	Location	Number of people with diabetes/ community members	Number of team members	Professional Roles	Number of team members from local community
Team West	Provincial and federal (combination)	Western Canada	134/2000	6	Family physician, Diabetes educator, Homecare nurse, Office assistant, Health promoter, Retinal photographer	3
Team East	Regional First Nations health board	Eastern Canada	300/2200	6	Family physicians (2), Registered nurse, Health service coordinator, Nutritionist, Community Health Representative	1

Team East included six team members including two family physicians, a health service coordinator, a nurse-in-charge, a nutritionist, and a community health representative. The clinic is located in a remote community, which is approximately 600 km from the nearest city with provincial health services. Table 3 provides a summary of the main and sub-themes. The findings include the common categories and themes from both communities, and where applicable unique community findings are highlighted.

**Table 3:** Summary of Themes and Sub-themes

Theme	Sub-Theme
1. Informing the Quality Improvement Process	Sharing of Perspective and Experiences Reflecting on Local Practice Information Integrating Evidence-based Practices Aligning Care to Client Needs and Culture
2. Strengthening and Building Relationships	Strengthening Relationships with Clients and Community Members Building Partnerships with the Community Team
3. Organizational Support and Capacity	Working within Existing Structures and Capacity Leadership and Team Support
4. Facilitating Quality Improvement	Engaging Team Discussion and Reflection Facilitating Learning

### Theme 1: Informing the quality improvement process

#### Sharing of perspectives and experiences

Members from both teams expressed that the sharing of perspectives and experiences among team members was important in enabling their process for improving diabetes care. In particular, reflecting on team members’ unique perspectives and experiences helped the teams build a better understanding of each other’s roles, enhanced their knowledge of resources available for people with diabetes, and helped to identify gaps or challenges to providing diabetes care. As described by one team member: *“I think it was helpful to have, you know, all those different inputs from the team members”* (Team West, Interview).

This sharing was facilitated by meeting as a team at the workshops and in the community. In addition, reviewing the clinical readiness report as a team and discussing everyone’s perspectives facilitated a more comprehensive understanding of the context for diabetes care in the community.

*The reports that we got back were helpful. So, it was worth it to go through [as a team] because you don’t always see the bigger picture. You just see what’s happening with you and then you get the answers of your team.* (Team West, Interview)

Team members shared how community members who were part of the teams provided further insight into their community’s culture and health practices, and offered a unique perspective on areas to target for QI and ideas for how to improve diabetes care.

*Just for the clinical team, I guess just having someone who lives in the community, works in the community, and raised in the community, just having that point of view or outlook on the ideas that were thrown around in the clinical team*. (Team West, Interview)

#### Reflecting on local practice information

Team members of both teams described how the Model for Improvement and PDSA cycles gave them a method to help identify problems within the system and areas of diabetes care they could focus on improving. It also provided a method to collect and evaluate information from within their practice to inform their diabetes QI activities.

*I think in the area of making changes, and evaluating them… and how we’re going to measure… previous to this, I don’t think we’d been doing any kind of evaluating [of] our programs. We would ask for some feedback, but otherwise, we weren’t really measuring or monitoring things like that, so I think we have a better capacity in evaluation.* (Team West, Interview)

Team members shared how they used the local practice and community information from the tools integrated in the program. Teams reflected on their clinical readiness report’s information to identify gaps in care and generate ideas for QI strategies: *“It made its differences. It identified what our starting lines are. And that kind of gave us an idea of where we’d like to go. And what would be achievable right now because of our isolation and all that”* (Team East, Interview).

Both teams used the diabetes registry and surveillance system or their existing charting system to help identify a list of clients with diabetes. However, the findings were mixed on whether the teams used these systems to inform their QI process. Team West discussed making better use of their electronic medical record to document clients’ clinical diabetes information and improve follow-up with clients. However, they did not specifically discuss using clinical information within the system to inform their QI work (i.e., identify gaps in care or evaluate the success of their strategies). Team East instead described using their existing system to show patients graphs of their recent blood work during clinical visits.

#### Integrating evidence-based practices

During interviews, team members discussed whether the workshops enhanced their overall knowledge of diabetes care: “*The training for me was really interesting to make sure that my knowledge was up-to-date. It made me more confident in my ability to help diabetic people”* (Team East, Interview). Furthermore, during team discussion at workshop breakout sessions, members expressed the importance of improving the frequency of client follow-up based on clinical guideline recommendations. They also developed strategies to improve care using ideas presented during the workshops, such as team-based care approaches, motivational interview techniques, and integrating discussion on spirituality during mental wellness visits.

#### Aligning care to client needs and culture

Building from concerns over low attendance to diabetes medical appointments or programs, both teams focused on developing strategies to reorganize care based on clients’ feedback on their preferences and needs for accessing services. For instance, Team East developed diabetes clinic days, where a dedicated team of nurses would provide care and follow-up to clients with diabetes. Diabetes clinic days were developed based on feedback from their clients and their wishes to see the same nurse at each visit to improve the continuity and consistency in the care they received.

*We’ve been struggling for so many years with [having] only one person, one health provider, to provide the care all the time with the same people. You know, you would come to the clinic, and you would ask for the nurse and that’s the nurse [you] would always see.* (Team East, Interview)

Another important aspect of Team East’s diabetes clinic days was adapting the frequency of the follow-up appointments based on clients’ preferences and schedules: “*Clients have preferences for how often they would like to come, which often do not align to when the clinical team wants them to come”* (Team East, observation field notes). Similarly, Team West restructured their existing weekly diabetes screening program by implementing joint, ad hoc appointments with the diabetes educator and family physician to improve appointment attendance.

The teams frequently drew upon their clients’ feedback when testing and adapting new QI strategies and evaluating their success. The value of client feedback is exemplified in the following quote when discussing the impact of Team East’s new QI strategies:

*We tried that way of working with only a small number of patients because it requires a high involvement from the patient to agree to come for many weeks in a row. And for the nurse also to commit to see those 10 patients regularly on top of their caseload also…. The patients that were followed closely they really appreciated that we did that together… I think it made a good difference that they were really taken care of.* (Team East, Interview)

In addition, team members discussed the importance of implementing QI strategies that aligned with the culture of the community. Both teams developed QI strategies that integrated the cultural traditions and community practices into evidence-based practices. For instance, Team West incorporated community traditions and a narrative approach in their group medical visit:

*I think in terms of the community and the narrative type of thing... it would be sitting in a circle and there can be a centrepiece… we’re in a circle and the facilitators are not lecturing, they’re not standing at the front of the room looking at them. I’m excited about those elements, you see it’s so culturally relevant, and especially when you are on a group visit and it’s like, well, the doctor’s health visit should be confidential, but it’s a community disease and that makes it look more like that we’re all in this together. You may have different manifestations from me but we’re all in this lifestyle community change together.* (Team West, Interview)

### Theme 2: Strengthening and building relationships

#### Strengthening relationships with clients and community members

An important part of the two teams’ diabetes QI activities was strengthening relationships and trust with their clients and the broader community to improve clients’ attendance appointments or participation in diabetes programs. Team West discussed how group visits and joint appointments with the diabetes educator and family physician were implemented to build relationships with clients and improve their likelihood of attending follow-up appointments with the diabetes educator. This was particularly important because the diabetes educator was new to working in the community. Similarly, Team East felt that implementing diabetes clinic days, where the client would see the same nurse at each visit, would not only improve consistency and continuity of care but also help to build relationships and trust with their clients.

Another important strategy that both teams used was increasing the team’s visibility and outreach in the community, outside of the health centre. Both teams felt that increasing their presence in the community would help them build relationships and trust with community members. One team member from Team West described the impact of their outreach strategies by offering healthy snacks at information booths at the band office:

*We’re definitely seeing more people coming in to get information, who are readier to change. It just seems like people are more open to it now… we’ve increased our presence in the community, so I’m hoping it’s because they now know us and we’re not just complete strangers.* (Team West, Interview)

#### Building partnerships with the community team

Throughout the QI program, both teams worked on building a partnership with the respective community teams with the goal of co-developing and implementing diabetes QI strategies. Team West found overlap in some of the diabetes education strategies they worked on. They described how it was easy for the clinical and community teams to connect because the members of both teams were located in the same building. One team member, who was a member of both the clinical and community team, described the benefit of having overlapping team membership: “*Even just a bit of an overlap of the two [teams] I feel is very helpful because you can talk separately about community and clinical but at the end of the day they kind of have to work together”* (Team West, Interview).

At the first workshop, Team East discussed the importance of working closely with the community team and receiving feedback from them on ideas they had for improving care. By working together, they felt it provided an opportunity for non-Indigenous team members to expand their understanding of the community’s culture and strengthen their relationship with the community. One member described their excitement of working with the community team:

*We were really excited to have the community team and the clinical team to get to meet each other and to – especially to get the comments from the community and to hear from them what they think about our delivery of services. We were really excited when we wanted to change the way we deliver the care to diabetic patients based on their needs… That was something I was really excited about. Tell us the way you want us to do it.* (Team East, Interview)

However, team members expressed difficulty regularly connecting and meeting with the community team due to scheduling conflicts and time constraints. Team members described feeling disappointed when meetings did not take place because they felt it was important that the community team help them adapt the way diabetes care is provided: *“we’re [from] different culture[s], you know, so we can’t just guess”* (Team East, Interview). Despite challenges, they recognized that building a partnership with them could take more time.

### Theme 3: Organizational support and capacity

#### Working within existing structures and capacity

Integral to the teams’ ability to implement changes in practice to improve diabetes care was having the organizational structure and capacity. Team members described how they focused their QI efforts on areas of diabetes care where they had the ability to make changes:

*The project was all about making it small. So, you know, we took the things that we knew we could make a difference on right away. And we just left aside the things that were too big and too expensive or, you know, impossible to clear right now, you know.* (Team East, Interview)

During workshop breakout sessions, team members often expressed their concerns with staffing issues and discussed how it was difficult to make changes and improve care without having people who were dedicated to diabetes care in the community, such as diabetes educators. They felt that staffing issues were out of their control and dependent on healthcare management and leadership to make changes. For Team West, this involved discussions early on in the program around the recent loss of the diabetes educator and they felt that client care had declined in their absence. Team East discussed challenges with some nurses’ limited knowledge or confidence in helping clients manage their diabetes.

*Well the high turnover of nurses made it really hard. It made it hard for us to do a good follow-up because the reality up north [is that] the nurses go on holidays and then the agencies that come to replace those nurses don’t necessarily have the knowledge or the confidence in taking care of diabetes patients… The nurses are here for eight weeks or so and then they leave for a month and they come back for eight weeks, they leave. So that was hard.* (Team East, Interview)

While feeling unable to make changes in some areas, teams were able to develop small changes with existing resources. To compensate for the absence of a diabetes educator on their team, Team West developed QI strategies that made better use of their electronic medical records and taught other healthcare professionals how to do foot examinations to improve follow-up on clinical practice guideline recommendations. Similarly, Team East tried to find ways to restructure care and work with available staffing to improve the team’s capacity to provide diabetes care. Their strategies included providing diabetes care training for community health representatives, developing diabetes clinic days, and creating diabetes templates for staff to use with clients.

The ability of team members to dedicate time to developing and testing QI strategies was a second critical component in facilitating practice changes. Some team members found it easier to participate when QI activities were an extension of their current role within the health centre.

*Probably what made it easy, is that, in the area of diabetes, that’s the program that I coordinate here at the health centre. So, you know, I could make time for it, and it – some of the things weren’t too far from my regular work, since we do some community events, and clinical work as well.* (Team West, Interview)

However, other team members found it difficult to test new QI strategies on top of their current workload and existing professional responsibilities.

*I think I didn’t have enough time to do everything we would like. Like we wanted to give out some tools for – to support the nurses when they were doing their follow up with clients. I didn’t have enough time to do that mainly because I have so many other things to do.* (Team East, Interview)

Members from Team East also described challenges with implementing practice changes because of an increase in emergencies in the health centre and continuing challenges with staffing support.

*We would try something new, like, every week and would keep track of the progress that we’ve made... But I think it was really an explosion, and the amount of emergencies that were showing up to the clinic and the severity of care that it would require…. And the staff has not much increased yet…But we have a lack of lodging, a lack of office space, a lack of staff. And then so we’re always, like, you know, pushing forward, you know, pushing things forward. So we just catch up and deal with emergencies. So that’s why it’s been so hard.* (Team East, Interview)

#### Leadership and team support

When asked what supported the development and implementation of changes in practice, team members described the importance of having the support from management and health directors. Additionally, having supportive, dedicated, and motivated members on the team was essential in maintaining momentum on their QI strategies and working together as a team.

*Sometimes it was hard to keep momentum going. So to keep having the meetings, and to keep thinking, okay, what else can we do… I think it really does take a team of, you know, motivated and dedicated people. And everyone’s so busy with all different kinds of schedules, that by the end, we were just kind of having meetings with whoever could come.* (Team West, Interview)

Team East described timing challenges with meeting as a team, but they found ways to ensure they continued to meet and maintain momentum on their strategies.

*The challenging part was you know, we’d get something going and then one or two or three of the members would either go on holidays or go on sick leave. But kind of [name of clinical team member] and I being the constants, were the ones that you know, tried to keep the meetings going where, you know, [other team members] phoned in from wherever they were and we just did some little PDSAs for each week and see how we met those.* (Team East, Interview)

### Theme 4: Facilitating quality improvement

#### Engaging team discussion and reflection

Program facilitators played key roles in aiding team discussion and reflection during workshop breakout sessions. The community facilitators guided the teams and kept their team members focused throughout the QI process, from identifying priority areas for improvement to developing specific QI strategies. When team members would get stuck on a particular idea, facilitators suggested the team step back and reflect on their roles and experiences to identify areas to target their QI activities. However, community facilitators sometimes had challenges in engaging the team in discussion and moving things forward. During the first workshop it was observed that community facilitators were not as confident in engaging team discussion, particularly when the teams started developing QI strategies and applying PDSA methodology. Additionally, community facilitators were sometimes inconsistent in asking for individual team member input and had challenges in ensuring all team members participated in the discussion. Western research team facilitators were able to support the community facilitator by providing encouragement and reminding them to seek the input from all team members.

#### Facilitating learning

The Western research team facilitators were instrumental in helping teams understand and apply the Model for Improvement and PDSA cycles throughout their participation in the program. Early in the program when teams were first starting to develop QI strategies, team members occasionally appeared uncertain on how to apply the methodology. Western research team facilitators helped the team set specific, feasible, and measurable goals, and, in particular, helped them through each step of developing and testing QI strategies using PDSA cycles:

*Well, it was nice to have people like you [research team] who were there, kind of guiding the discussion. And especially when these PDSAs are- and you’re trying to wrap your head around them and not making them too big and small and you have chunks to be able to cycle them through. So that was really useful, to have somebody being there to do that and guide us and redirect us.* (Team West, Interview)

During end-of-program interviews, many team members described how the Model for Improvement and the PDSA cycles had become “second nature.” One team member further described how these QI methods changed their way of thinking:

*It changed our mindset, completely. But, at the end of the day, the PDSA tool that you gave us, the way we changed our minds when facing a problem, I mean, it’s priceless … I mean, we’re using it every day… with anything that happens*. (Team East, Interview)

## Discussion

Closing gaps in health outcomes between Indigenous and non-Indigenous populations and improving health care delivery in First Nations communities in Canada is a national priority [34]. Research is needed on the strategies that can improve the quality of care provided to First Nations peoples with diabetes in Canada and address the inequities in access to culturally appropriate and optimal care. This study contributes to the research base on QIC programs by generating an in-depth understanding of the development and implementation of diabetes QI strategies, the underlying knowledge exchange process, and how QI can be supported and facilitated in First Nations communities in Canada. Understanding the QI process occurring across diverse contexts for primary care delivery in First Nations communities in Canada can help inform policy and future development, implementation and scale-up of QIC programs.

The FORGE AHEAD clinical-based QIC program demonstrated the potential to improve access to quality care and improve the health and well-being of First Nations peoples living with diabetes. The Model for Improvement and PDSA cycle methodology provided clinical teams with a method for problem-solving local challenges to the provision of diabetes care in their communities. Team members from the two teams described how it gave them a method for identifying problems and setting QI goals, and enhanced skills to collect and evaluate information from within their practice. Importantly, the program shows potential for facilitating an environment where the organization has the skill set, confidence, and mindset to test, implement, and evaluate changes in practice to improve the quality of care provided to people living with diabetes in First Nations communities in Canada. The literature shows that a shared and collective commitment to change and the capability to do so are considered key elements to the successful implementation of complex changes in health care settings [35].

In the prevention and management of diabetes complications, evidence-based practice is usually emphasized within regards to the use of clinical practice guidelines. However, it is extensively argued in the literature that both explicit (in the form of research evidence and guidelines) and tacit knowledge (in the form of experiential and context-specific evidence of the broader environment) play a role in decision-making and have a mutual and supporting role with each other [36]. The findings of this study showed how these two clinical teams used both explicit and tacit knowledge to inform decisions on the development and implementation of changes in practice to improve diabetes care. Similar to other studies [37], tacit knowledge appeared to play a greater role in the teams’ collective decision-making and planning throughout their QI process.

Tacit knowledge utilized by the teams included the perspectives and experiences of team members, clients with diabetes, and community members, as well as local practice knowledge. The findings demonstrated how meeting as team at the workshops, as well as back in the community, facilitated the sharing of various team members’ clinical experiences and enabled the teams’ QI process. The sharing of diverse team members’ perspectives was facilitated through the process where teams’ discussed their team’s clinical readiness report. Tools such as this can help formalize a process for capturing, merging, and sharing of both tacit and research-based knowledge of optimal approaches to diabetes care [38]. Moreover, these two teams relied on client feedback to measure the impact of their QI strategies more so than measuring objective, clinical performance data that is traditionally emphasized in QIC programs. These findings are consistent with those of Farr and Cressey [39], who found that healthcare professionals’ perceptions of the quality of practice relied upon relational and tacit dimensions of care, including their values, motivations, and behaviours, and interactions with patients. Similarly, other studies evaluating QIC programs showed that healthcare professionals wished there was less focus on clinical outcomes [40].

Unique to the context of QI in First Nations communities, this study demonstrated the importance of community members’ perspectives in informing the teams’ QI strategies. Community members provided insights into the community’s culture and barriers to accessing care, as well as shared ways to improve diabetes care. Our findings highlighted how some non-Indigenous healthcare professionals may be separated from the broader community and have a limited understanding of the community’s culture. These findings have potentially important implications for both practice and QI programs in First Nations healthcare settings that may be considered for similar programs.

Drawing upon the tacit knowledge of community members and their understanding of how and why things ‘are the way they are’ can help healthcare professionals identify essential factors for the successful implementation of health services and programs [41, 42]. Non-Indigenous healthcare professionals’ recognition of local Indigenous knowledge and actions taken to improve understanding of the community can reduce the colonial history of health care services and improve access to care [42, 43]. The development of respectful and trusting partnerships between healthcare professionals and the broader community in the design and implementation of health care services can ensure that services are respectful of the local culture and traditions [41, 44]. It is also important to emphasize the participation of Indigenous healthcare professionals on the QI team. They can act as cultural mentors for non-Indigenous staff and assist in the provision of culturally appropriate and safe health services [41].

Within First Nations health care settings, where healthcare professionals from diverse cultural backgrounds are collaboratively solving problems in primary and diabetes care, it will require strategies that enable an ethical and safe space for sharing knowledge and perspectives [45]. Although a community facilitator may theoretically be an ideal person to facilitate daily practice dialogue and interaction among the team, our findings from these two teams showed that they were not always able to effectively facilitate team discussion or ensure all team members’ voices around the table were heard. Through experience and training, studies have shown that external practice facilitators are effective at encouraging team member involvement and collective decision-making within existing professional hierarchies [46]. However, the community facilitator may play an important role in sustaining team-based reflection and sharing after the support of external facilitators is ceased upon completion of the formal QIC program. Through enhanced training on facilitating team dialogue and handling group dynamics, literature shows that internal community facilitators can play an effective role in daily practice dialogue and flattening professional hierarchies through the empowerment of a space for all team members’ voices to be heard, which, in turn, can help establish and maintain a culture for QI [47].

Consistent with other studies, our findings showed key factors to supporting QI in primary care settings, such as leadership support and time available to dedicate to QI work [48–50]. However, our findings demonstrate system-level factors unique to these two First Nations communities that hindered primary healthcare teams’ ability to improve diabetes care. For example, our findings showed challenges in providing consistency and continuity of diabetes care due to ongoing staff shortages and high turnover within the communities. Studies have shown that shortages of healthcare professionals and high turnover within First Nations communities, especially in remote and isolated communities, creates challenges in building and nurturing trustful relationships with First Nations clients [51–53]. Systemic-level and workforce barriers to diabetes care in First Nations communities have hindered primary healthcare professionals’ ability to best support people living with diabetes [51, 54, 55]. In a recent study by Crowshoe et al. [51], family physicians and specialists who provide care in First Nations communities in Canada described how they felt powerless to transform service. Our findings demonstrated that even in the presence of structural and policy challenges, teams were motivated to change and implement small changes within areas of care where they felt they could make a difference. However, these system-level challenges may jeopardize the ability to see sustained improvements in care. This suggests the need for policy reform and infrastructure support to adequately address issues with access to quality care and improve diabetes health outcomes in First Nations communities in Canada.

### Recommendations for quality improvement collaborative programs

Future QIC programs may want to find a balance between providing “new” knowledge at workshops to improve diabetes care and providing opportunities for team-based sharing in the creation of knowledge for QI. This may include the creation of tools to capture and amplify the experiential knowledge of individuals within the healthcare settings and from community members. Furthermore, programs could look at ways to promote and support more opportunities for networking with other teams to learn about similar experiences, challenges, and ideas for QI strategies during and after the program. One potential alternative knowledge exchange strategy may be virtual communities of practice.

Lastly, the finding showed that the importance of supporting continual partnerships and knowledge sharing between healthcare professionals and community members. Future programs and research may seek to explore strategies to support social and cultural aspects of health and more community engagement within First Nations primary healthcare clinics. Strategies may include: community events that promote community relationship building; interventions centred on self-determination and local priorities; interventions that educate healthcare providers to re-centre relationship building with their clients and cultural engagement; and demonstrating ways to support traditional practices within healthcare plans [56].

### Study strengths and limitations

With limited knowledge on the implementation processes occurring within QIC programs, this study provided an in-depth understanding of diabetes QI activities occurring across diverse primary care contexts in two First Nations communities. Importantly, these findings identify factors important for the sustainability of QI and chronic disease care, and generates insights for future research, policy, and programs. This study illustrated how a QIC program might facilitate the improvements in access and quality of care in First Nations communities in Canada, and the factors that can support QI in these settings.

Community representatives were continually involved in conversations around the scope of this research, ensuring that the study findings would generate knowledge beneficial to the community. Community representatives were also involved in the interpretation of the initial findings to obtain valuable insight into the data from those directly involved in QI strategies within the community. Using multiple data collection methods and involving First Nations community representatives and researchers from diverse disciplinary backgrounds enhanced the credibility of the findings and provided a deeper understanding of the teams’ QI processes.

This study utilized case study methodology with two clinical teams from First Nations communities that participated in the FORGE AHEAD clinical-based QIC Program. The findings in this study were generated from a small number of participants, which has limited generalizability [57]. However, case study methodology, and qualitative research more generally, focuses more on the particularization and contextualization of research findings rather than generalization [29]. The study findings attend to complexities of improving diabetes care as they are situated within two particular First Nations communities and primary care contexts, from which may inform and provide insight for the development of future research, programs, and policies.

There are inherent limitations to the use of secondary data sources in qualitative research. The use of various forms of knowledge and the perceived value of this knowledge for informing the teams’ diabetes QI processes emerged in interviews. Although it would enrich the findings, participants were not explicitly asked in the interviews about the use and value of various forms of knowledge, such as research evidence or tacit knowledge. Secondly, the use of secondary data sources in qualitative research precluded simultaneous data collection and analysis and removed the ability to return to participants to further explore emerging findings and deeper meaning [58]. This study’s findings would have been enriched by exploring emerging themes from data collected throughout the program, such as observational or implementation support notes, in addition to end-of-program team member interviews.

## Conclusions

The findings of this study begin to paint a picture for understanding the process of developing and implementing changes in practice to improve the quality of diabetes care in First Nations communities in Canada. Future research may explore some of the emerging themes from this study, including how various forms of knowledge are negotiated and integrated to inform QI activities, how Western and Indigenous knowledge systems come together to inform clinical practice and change, and strategies that can help facilitate an ethical and safe space for doing so. Teams described how the Model for Improvement and PDSA methodology changed their way of thinking, demonstrating its importance in sustaining practices change and continuous quality improvement. Additionally, research may further explore the role of program facilitators and the sustainability of QI in communities and how community participation raises awareness of inequities and promotes advocacy for change.

## Data Availability

The datasets generated during and/or analyzed during the current study are not publicly available to respect the wishes of our partnering First Nations communities and individual participants but are available from the corresponding author on reasonable request and approval from partnering communities and individual participants.

## Abbreviations

FORGE AHEAD: TransFORmation of IndiGEnous PrimAry HEAlthcare Delivery
PARIHS: Promoting Action on Research Implementation in Health Services Framework
PDSA: Plan-Do-Study-Act
QI: Quality Improvement
QIC: Quality Improvement Collaborative
